# Drug-Drug Interactions in Nursing Home Residents With vs Without Dementia

**DOI:** 10.1016/j.jamda.2026.106417

**Published:** 2026-08-11

**Authors:** Mallory Go, Laura A. Reich, Daniel A. Harris, Lori.A. Daiello, Sarah D. Berry, Adam M. D’Amico, Andrew R. Zullo, Kaleen N. Hayes

**Affiliations:** a Department of Epidemiology, Brown University School of Public Health, Providence, RI, USA; b Center for Gerontology and Healthcare Research, Brown University School of Public Health, Providence, RI, USA; c Department of Health Services, Policy and Practice, Brown University School of Public Health, Providence, RI, USA; d Department of Epidemiology, College of Health Sciences, University of Delaware, Newark, DE, USA; e Department of Neurology, Alpert Medical School of Brown University, Providence, RI, USA; f Hinda and Arthur Marcus Institute for Aging Research and Department of Medicine, Hebrew SeniorLife, Boston, MA, USA

**Keywords:** Drug-drug interactions, older adults, nursing home, dementia, Alzheimer’s disease

## Abstract

**Objectives:**

To examine whether nursing home (NH) residents with Alzheimer’s disease and related dementias (ADRD) are more likely than those without ADRD to be exposed to potential drug-drug interactions (DDIs) and to experience longer durations of exposure to DDIs.

**Design:**

Retrospective observational study.

**Setting and Participants:**

Long-stay US NH residents.

**Methods:**

We leveraged Medicare Fee-for-Service enrollment and claims data linked to Minimum Data Set 3.0 clinical assessments from 2018 to 2020 and identified NH residents aged ≥66 years with observable part D prescription drug data. We identified exposure to 98 potential DDIs during the duration of their NH stay. We described the number of DDIs with a clinically meaningful difference in prevalence and/or duration between groups, defined as a ≥1% difference in prevalence and ≥7 days’ duration, respectively. We used a log-binomial model to estimate the association between ADRD and exposure to any DDI as an adjusted prevalence ratio with 95% CIs and predicted the marginal prevalence of exposure to any DDI for those with vs without ADRD, standardized to population covariate distributions.

**Result:**

We identified 485,251 eligible NH residents (average age, 84.6 [SD, 8.1] years; 67.1% female; 70.1% with ADRD; 56.6% with a potential DDI). A diagnosis of ADRD was associated with a higher prevalence of DDI exposure (prevalence ratio, 1.09 [95% CI, 1.08, 1.09]; marginal predicted prevalence for those with vs without ADRD: 58.0% vs 53.3%). Twelve DDIs had different prevalences between groups, and 34 DDIs had different differences in duration. For example, 29.6% of residents with vs 21.2% without ADRD were exposed to ≥3 central nervous system—active drugs.

**Conclusions and Implications:**

NH residents with ADRD had a higher prevalence and duration of potential DDIs. Future research should evaluate whether the interactions we identified have causal effects on adverse outcomes such as falls.

Alzheimer’s disease and related dementias (ADRDs) affect over 5 million Americans.^1–3^ Due to the extent of multimorbidity in this population, approximately 50% of individuals with ADRD have polypharmacy (ie, the use of 5 or more concurrent prescription medications).^3^ Older adults in nursing homes (NH) have an even higher medication burden, with 65% receiving 10 or more prescription drugs.^3–5^ Adverse drug events, especially those caused by drug-drug interactions (DDIs), are of particular concern for NH residents given the degree of polypharmacy.^6^ Blood-brain barrier dysfunction and changes in neurotransmitter systems are features of ADRD that may heighten sensitivity to the adverse effects of psychoactive medications,^7,8^ and age-related changes in hepatic metabolism, renal clearance, and reduced expression or activity of some drug transporters and metabolizing enzymes may further amplify the risk of pharmacokinetic and pharmacodynamic DDIs in ADRD.^7,8^ Due to these factors, people living with ADRD may be more likely to experience adverse effects from DDIs.^1,5,9,10^ However, no population-based study has described differences in the prevalence of DDIs between NH residents with and without ADRD. Understanding any DDI-specific differences can guide prescribers, pharmacists, and policymakers to develop tailored deprescribing strategies, medication review protocols, and risk mitigation efforts that account for an individual’s cognitive status.^10^ In this study, our objective was to compare the prevalence and duration of exposure to potentially clinically significant DDIs between NH residents with and without ADRD.

## Methods

### Study Design, Data Sources, and Study Population

This retrospective cohort study examined US NH residents using linked data from the Minimum Data Set (MDS) 3.0 (mandated clinical assessments performed at least quarterly^11^) and Medicare enrollment and claims data from 2017 to 2020.^11^ The study included Medicare Fee-for-Service beneficiaries aged ≥66 years with at least 12 months of continuous enrollment in Medicare parts A, B, and D before each NH stay, and no enrollment in Medicare Advantage.^12^ Because of bundled payment structures that prohibit part D claims during post-acute care stays, NH stays were defined as continuous periods of residence in an NH during which part D prescription drug dispensing data were observable (ie, after any period of post-acute care ended), as defined by a published algorithm.^13^ Stays began on the first day of NH entry or re-entry not covered by Medicare part A’s post-acute skilled nursing benefit and ended at the earliest of the following: disenrollment from Medicare parts A, B, D, or transition to Medicare Advantage; part A post-acute care or hospitalization; NH discharge; death; or study end (December31, 2020). Beneficiaries could contribute multiple eligible NH stays.

### Identification of Potential DDIs

Potential DDIs were identified using a list of 98 clinically important DDIs compiled from 3 sources that used structured expert consensus methods: Anrys et al,^14^ the 2023 American Geriatrics Society Beers Criteria for Potentially Inappropriate Medication Use in Older Adults,^15^ and Capiau et al.^16^ The 2023 American Geriatrics Society Beers Criteria^15^ and Anrys et al^14^ identified DDIs relevant to older adults, and Capiau et al^16^ focused on interactions involving direct oral anticoagulants, which are increasingly prescribed to NH residents in the United States.^17^ Individual DDIs are referred to in quotations as they are specified in each respective list. Methods used to operationalize DDIs in the Medicare claims data have been described previously.^12^ In brief, orally administered drug pairs were considered concurrently used if there was at least 1 day of overlapping supply during the NH stay as identified via part D claims. The primary outcomes included (1) the prevalence of exposure to each potential DDI during the NH stay and (2) the duration of exposure, measured as the number of overlapping days for each DDI pair, allowing for nonconsecutive days of overlap.

### Resident Characteristics

For residents who contributed multiple eligible NH stays, the first NH stay was used to measure characteristics. Diagnosis of ADRD was identified using the first documented date from the MBSF 27 Chronic Conditions Warehouse algorithm, with individuals classified as having ADRD if they had a record of a diagnosis before their first NH stay.^18^ Resident demographics were obtained from the Medicare Beneficiary Summary File.^19^ Clinical characteristics were derived from the MDS and the Medicare Provider Analysis and Review file (similar to part A claims). Multimorbidity burden was quantified using the combined comorbidity score based on Medicare Provider Analysis and Review claims in the 12 months preceding the NH stay.^20^ Cognitive function was measured using the Cognitive Function Scale from the most proximal MDS assessment prior to the start of follow-up.^21^

### Statistical Analysis

We used absolute standardized mean differences to compare characteristics between residents with and without ADRD.^22^ We estimated 95% CIs for DDI prevalence estimates using the Clopper-Pearson exact method. We compared the duration of each DDI exposure as the difference in the median days exposed among those with at least 1 day of exposure. Based on clinical input and the preliminary distribution of results, we considered an absolute difference in prevalence of ≥1% and a median duration of ≥7 days to be clinically meaningful. To contextualize the difference in prevalence for each DDI, we then multiplied the difference in prevalence by the number of residents with ADRD to estimate the excess number of residents with ADRD exposed to each DDI.

We fit a log-binomial model to examine the association between ADRD and exposure to any DDI as a prevalence ratio with 95% CI, adjusting for resident age, sex, race/ethnicity, and combined comorbidity score.^23,24^ Only the first eligible NH stay was included in this model. We opted for parsimonious adjustment of models to avoid accounting for mediators of the relationship between ADRD and potential DDI exposure, such as cognitive function. We constructed 4 log-binomial models with varying covariate adjustment and spline terms to account for potential nonlinearity, selecting the best-fitting model based on the lowest Akaike information criterion.^25^ We then used the best-fitting model to estimate the predicted probability of exposure to a potential DDI by ADRD diagnosis, marginalized over the population distribution of covariates. This study was approved by the Brown University Institutional Review Board, which waived the requirement for informed consent. All analyses were conducted using SAS, version 9.4 (SAS Institute, Inc) and R (version 2025.05.1+513), and relevant code is publicly available.^26^

## Results

We identified 485,251 eligible residents (70.1% with ADRD; average age, 84.6 (SD, 8.1) years, 67.1% female; 84.2% White; Table 1) who contributed 709,330 stays. An exclusion flow diagram is available in Supplementary Figure 1. More than half of all residents (56.6%) were exposed to at least 1 potential DDI. The most common potential DDIs were “concomitant use of ≥3 central nervous system (CNS)—active drugs,”^14^ “any combination of ≥3 CNS-active drugs,”^15^ and “selective serotonin reuptake inhibitors (SSRIs) plus another serotonergic drug (including tramadol).”^14^

Residents with ADRD had a higher unadjusted prevalence of exposure to any DDI (57.7%) compared with those without ADRD (54.1%). The best-fitting model (Table 2) estimated a 9% increased relative prevalence of DDIs among those with ADRD vs those without (prevalence ratio, 1.09; 95% CI, 1.08-1.09), and the marginal predicted prevalence of any DDI exposure after standardizing for covariates was 58.0% for residents with ADRD and 53.3% for those without ADRD (prevalence difference, 4.7%; number of excess residents with ADRD exposed, 15,981).

**Fig. 1.**
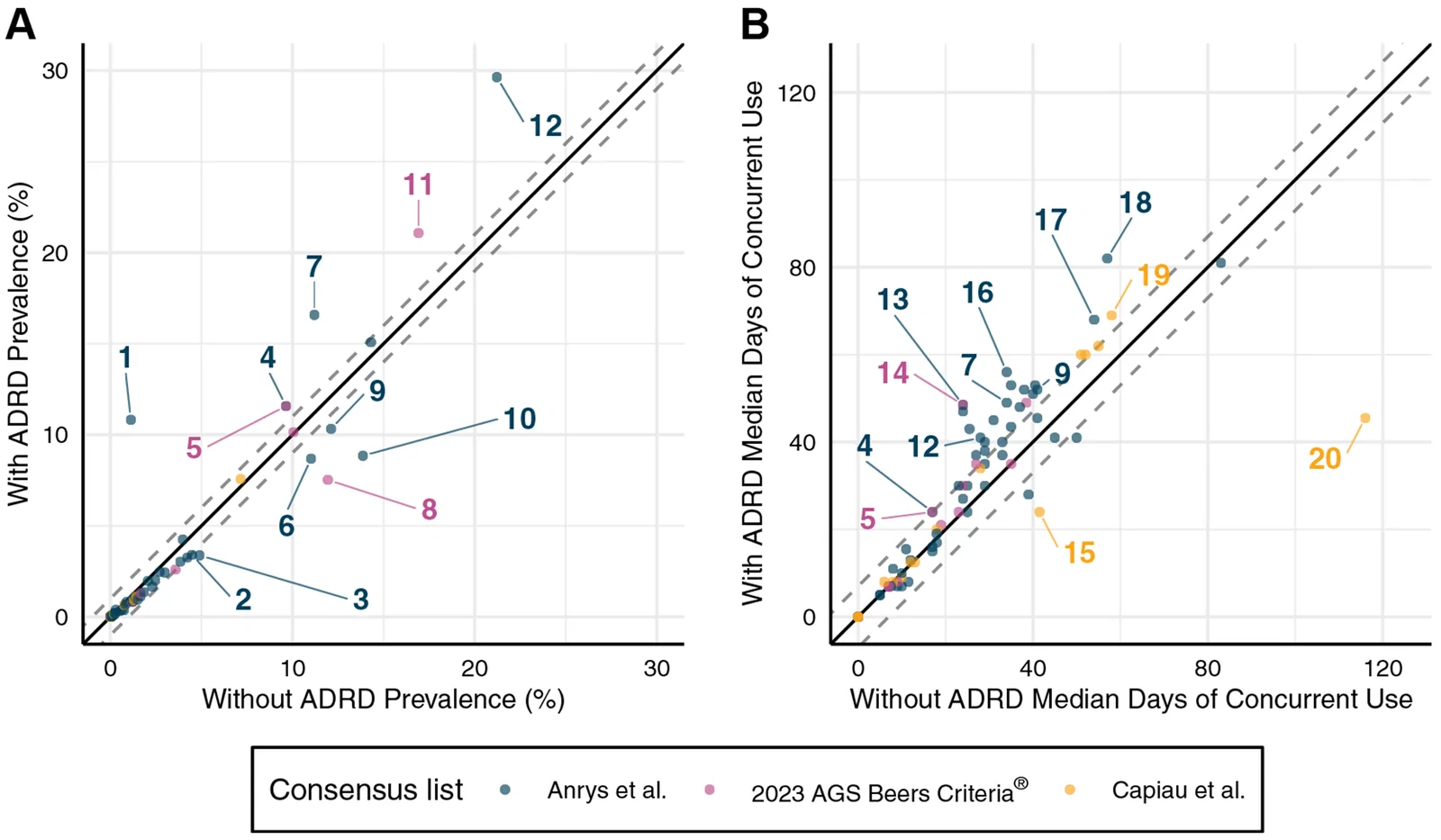
Prevalence (A) and duration of exposure (B) to potential DDIs by ADRD status. Labeled points: (1) Acetylcholinesterase inhibitor + a drug that reduces heart rate (antiarrhythmic drugs, beta-blockers, verapamil, or diltiazem) (Anrys); (2) ACE inhibitor or ARB + an oral NSAID (Anrys); (3) diuretic + oral NSAID (Anrys); (4) concomitant prescription of ≥2 anticholinergic drugs (Anrys); (5) concomitant use of ≥2 medications with anticholinergic properties (Beers); (6) concomitant use of ≥2 potassium-sparing drugs (Anrys); (7) SSRI + another serotonergic drug (including tramadol) (Anrys); (8) opioids + gabapentin or pregabalin (Beers); (9) ACE inhibitor or ARB or a potassium-sparing diuretic + a potassium supplement (Anrys); (10) concomitant prescription of ≥2 drugs that reduce potassium (Anrys); (11) any combination of ≥3 CNS-active drugs (Beers); (12) concomitant use of ≥3 CNS-active drugs (Anrys); (13) lithium + ACE inhibitor or an ARB (Anrys); (14) lithium + ACEI or ARBs or ARNIs (Beers); (15) verapamil + dabigatran (Capiau); (16) digoxin + verapamil or diltiazem (Anrys); (17) SSRI + loop or thiazide diuretic (Anrys); (18) tamoxifen + citalopram or escitalopram (Anrys); (19) SSRIs or SNRIs + direct oral anticoagulants (apixaban, dabigatran, edoxaban, or rivaroxaban) (Capiau); (20) phenobarbital + direct oral anticoagulants (apixaban, dabigatran, edoxaban, or rivaroxaban) (Capiau). Each point represents a potential DDI from the sourced consensus list: Anrys et al^14^ (blue), the 2023 AGS Beers Criteria (pink), and Capiau et al^16^ (yellow). Points labeled 1 to 12 in panel A denote potential DDIs with a meaningful difference in prevalence (≥1%) by ADRD status. Labeled points in panel B denote a selection of potential DDIs with a meaningful difference in median duration (≥7 days) by ADRD status. The solid black line indicates no difference in prevalence or median duration, and the gray dashed lines indicate the threshold of meaningful difference. ACE, angiotensin-converting enzyme; ACEI, angiotensin-converting enzyme inhibitor; ADRD, Alzheimer’s disease and related dementia; AGS, American Geriatrics Society; ARB, angiotensin receptor blocker; ARNI, angiotensin receptor/neprilysin inhibitor; NSAID, nonsteroidal anti-inflammatory drug; SNRI, serotonin and norepinephrine reuptake inhibitor.

Differences in DDI prevalence and median duration between residents with and without ADRD are shown in Figure 1. Twelve DDIs had meaningful differences in prevalence, and 34 DDIs had meaningful differences in median duration between those with and without ADRD. Comprehensive prevalence estimates for all potential DDIs, including the number of excess residents with ADRD exposed to each DDI, are presented in Supplementary Tables 2–4. For example, 29.6% of residents with ADRD were exposed to concomitant use of ≥3 CNS-active drugs vs 21.2% prevalence among those without ADRD.^14^ Similarly, the combination of “SSRI + another serotonergic drug (including tramadol)” was found in 16.6% of residents with ADRD, compared with 11.2% in those without ADRD.^14^ Conversely, certain DDIs, such as “concomitant prescription of ≥2 drugs that reduce potassium,” were less common among residents with ADRD (8.9% vs 13.9%) compared with residents without ADRD.^14^

Among those with at least 1 day of potential DDI exposure, residents with ADRD had longer median DDI durations for several potential DDIs. Supplementary Tables 5–7 present durations of exposure. For example, the median duration of exposure to “concomitant use of ≥3 CNS-active drugs” was 41 days in residents with ADRD, compared with 28 days in residents without ADRD.^14^ Likewise, median exposure was notably longer for an “SSRI + another serotonergic drug (including tramadol)” (49 vs 34 days) and “concomitant use of ≥2 medications with anticholinergic properties” (24 vs 17 days).^14^ Although exposure to a “beta-blocker + verapamil or diltiazem”^14^ had a less than 1% difference in prevalence between groups, the median duration of exposure to a beta-blocker and verapamil or diltiazem among residents with ADRD was 52 days, compared with 38 days in residents without ADRD (a difference of 14 days).^14^

## Discussion

In this large national cohort of older long-term NH residents, we found that potential DDI exposure affected more than half of all residents. Residents with ADRD were particularly vulnerable, with 4.7% more having a potential DDI exposure compared with those without ADRD, even after standardizing for age, sex, and comorbidity burden. With around 1.2 million NH residents in the United States, this higher prevalence may translate to an excess of nearly 40,000 more NH residents with ADRD being exposed to a DDI among a population already at extremely high risk of polypharmacy and DDIs.^27^

Polypharmacy involving CNS-active drugs was both more common and of longer duration in residents with ADRD.^28–30^ This is particularly concerning, given the increased sensitivity of individuals with ADRD to CNS-active agents and their associated risks, including falls, sedation, and further cognitive decline.^2,28,30^ Similar patterns were observed for serotonergic combinations and anticholinergic burden, all of which are well documented in geriatric prescribing guidelines as high-risk combinations.^31–35^ These higher prevalences could be driven by a combination of greater multimorbidity and potential overreliance on medications for management of psychiatric and behavioral symptoms in long-term care settings.^36^

Certain DDIs, such as those involving potassium-reducing agents, were less common among residents with ADRD. This lower prevalence may reflect differential prescribing practices or clinical priorities in managing patients with cognitive impairment.^37,38^ For example, potassium-sparing regimens are often perceived as high-risk and high-maintenance therapies because of a potential risk of acute kidney injury, hyperkalemia, hypotension, and falls. Their use thus commonly requires monitoring of potassium levels via frequent laboratory measures. Providers in NH may therefore focus on deintensification of potassium-modifying regimens to reduce the need for frequent monitoring among individuals living with ADRD. Moreover, while some DDIs showed comparable prevalence across groups, the durations of exposure were significantly longer in those with ADRD, suggesting either delays in deprescribing or ongoing need for treatment despite interaction risk.^10,28,36^ Current research has cited clinical inertia and the many medications associated with multimorbidity in those with ADRD as potential reasons for the increased prevalence and duration of DDIs.^10,23,32^

This study has several limitations. Our analysis relied on administrative claims data, which does not capture most over-the-counter medications or clinical decision-making context, and does not enable us to distinguish pro re nata (as needed) use or actual ingestion. Although we were not aiming to generate causal estimands in this study, additional covariates may be of interest for studies examining the effects of complex prescribing decisions in end-of-life or palliative care settings.^5^ In addition, other resident characteristics, such as gender, race, and age, as well as facility-level characteristics (eg, rurality or ownership status), may be important sources of variation in prescribing patterns and potential DDIs for future research. Finally, we leveraged the Medicare Chronic Conditions Warehouse definition of dementia, which is highly specific (78%-92%) but has lower sensitivity (57%-64%) and may result in false negatives.^18,39,40^ Nevertheless, this measure identified a group of residents who appeared to be at higher risk of exposure to potential DDIs.

## Conclusions and Implications

Potentially harmful DDIs are common among NH residents, with disproportionately higher prevalence and often longer duration among those with ADRD. Our findings highlight the need for targeted medication safety interventions in this vulnerable population, especially regarding CNS-active and serotonergic drug combinations.^28^ Future research should explore the clinical consequences of these potential DDIs and evaluate the effectiveness of deprescribing strategies and decision-support tools tailored to the cognitive status of older adults.^6^ Reducing DDIs may represent a critical step toward improving quality of care and patient safety in long-term care settings.^28^

## Supplementary Material

Supplementary File

Supplementary data related to this article can be found online at https://doi.org/10.1016/j.jamda.2026.106417.

## Figures and Tables

**Table 1:** Characteristics of NH Residents by Diagnosis of ADRDs, 2018 to 2020 (N = 485,251)

Characteristic	Overall N = 485,251	Without ADRD n = 145,227	With ADRD^*^ n = 340,024	aSMD

	N (%)			
Age in y, mean (SD)	84.55 (8.10)	82.92 (8.69)	85.25 (7.74)	0.28
Female sex	325,845 (67.10)	95,569 (65.80)	230,276 (67.70)	0.04
Race/ethnicity				0.12
White	408,785 (84.20)	126,073 (86.80)	282,712 (83.10)	
Black	36,917 (7.60)	8948 (6.20)	27,969 (8.20)	
Hispanic	21,361 (4.40)	4878 (3.40)	16,483 (4.80)	
Asian/Pacific Islander	11,514 (2.40)	2979 (2.10)	8535 (2.50)	
Unknown/Other^†^	6674 (1.40)	2349 (1.60)	4325 (1.30)	
Combined comorbidity score, mean (SD)^‡^	4.78 (3.80)	4.24 (3.80)	5.01 (3.78)	0.21
Cognitive function^§^				0.87
Missing	78,680 (16.20)	30,130 (20.70)	48,550 (14.30)	
Cognitively intact	167,044 (34.40)	79,395 (54.70)	87,649 (25.80)	
Mildly impaired	106,779 (22.00)	25,380 (17.50)	81,399 (23.90)	
Moderately impaired	108,961 (22.50)	8534 (5.90)	100,427 (29.50)	
Severely impaired	23,787 (4.90)	1788 (1.20)	21,999 (6.50)	
Diagnosis of chronic kidney disease prior to the NH stay*	328,319 (67.70)	96,605 (66.50)	231,714 (68.10)	0.04
Number of unique orally administered active drug ingredients dispensed during NH stay, mean (SD)	6.71 (5.41)	6.28 (5.48)	6.89 (5.36)	0.11
Length of NH stay, days, median (Q1, Q3)	54 (15, 199)	35 (13, 138)	66 (17, 225)	0.20
Exposure to ≥1 potential DDI during NH stay	274,603 (56.60)	78,514 (54.10)	196,089 (57.70)	0.07

aSMD, absolute standardized mean difference (between those with vs without ADRD); Q1, first quartile; Q3, third quartile.

Characteristics were ascertained for each resident based on their first NH stay and are reported as the number (%) unless otherwise labeled. Covariates with missing values were retained and reported as missing in descriptive summaries.

* Based on first occurrence dates identified by the 27 Chronic Conditions Warehouse algorithm.

† Includes categories unknown, other, and American Indian/Alaska Native.

‡ Scores range from −2 to 26, with higher values indicating greater multimorbidity.

§ Measured using the MDS Cognitive Function Scale.

**Table 2:** Results From Log-Binomial Models Estimating Association Between ADRD (vs No ADRD) and Prevalence of DDIs During Their First Eligible NH Stay (N = 485,251)

Resident Characteristics	Model 1 (Base-Adjusted)	Model 4 (Best-Fit Based on AIC)
	PR (95% CI)	
ADRD (vs without ADRD)	1.09 (1.08, 1.10)	1.09 (1.08, 1.09)
Female sex (vs male)	1.18 (1.17, 1.18)	1.18 (1.17, 1.18)
Race/ethnicity: Asian/Pacific Islander (vs White)	0.69 (0.67, 0.71)	0.69 (0.68, 0.71)
Race/ethnicity: Black (vs White)	0.79 (0.79, 0.80)	0.80 (0.79, 0.81)
Race/ethnicity: Hispanic (vs White)	0.86 (0.85, 0.87)	0.87 (0.85, 0.88)
Race/ethnicity: Unknown/Other (vs White)	0.86 (0.84, 0.88)	0.87 (0.85, 0.89)
Age at start of NH stay (per 5 y)	0.97 (0.97, 0.97)	—
Combined comorbidity score (per 1-unit increase)	1.00 (1.00, 1.00)	—
Age at start of NH stay (linear spline term)	—	1.00 (0.99, 1.00)
Age at start of NH stay (restricted cubic spline term 2)	—	0.99 (0.98, 1.00)
Age at start of NH stay (restricted cubic spline term 3)	—	0.81 (0.76, 0.87)
Combined comorbidity score (linear spline term)	—	0.97 (0.96, 0.97)
Combined comorbidity score (restricted cubic spline term 2)	—	1.21 (1.19, 1.23)
Combined comorbidity score (restricted cubic spline term 3)	—	0.62 (0.60, 0.65)

AIC, Akaike information criterion; PR, prevalence ratio.

Table 2 presents PRs and 95% CIs from log-binomial models evaluating the association between ADRD and exposure to any DDI, adjusting for resident age, sex, race/ethnicity, and combined comorbidity score.

NB: Results from models 2 and 3 are presented in the supplement.
