# Supplementary material for: Drug-Drug Interactions in Nursing Home Residents With vs Without Dementia
Source: J Am Med Dir Assoc. Author manuscript; Available in PMC 2026 Sep 3. (PMC13537206; doi:10.1016/j.jamda.2026.106417)
Supplement: Supplementary file 1 [file NIHMS2203754-supplement-Supplementary_File.docx]

**Supplementary Material**

*Drug-drug interactions in nursing home residents with vs without dementia*

**Supplemental Figure 1.** Flow diagram of individuals included in the analytic sample.

**Supplemental Table 1.** Sequentially adjusted model results for prevalence ratio of potential DDIs between NH stays for individuals living with ADRD vs no ADRD (N=485,251 eligible NH-stays).

**Supplemental Table 2.** Potential Drug Interactions Among Nursing Home Residents (Anrys et al.), by ADRD Status, 2018-2020

**Supplemental Table 3.** Potential Drug Interactions Among Nursing Home Residents (Beers et al.), by ADRD Status, 2018-2020

**Supplemental Table 4.** Potential Drug Interactions Among Nursing Home Residents (Capiau et al.), by ADRD Status, 2018-202

**Supplemental Table 5.** Duration of Exposure to Potential Drug Interactions Among Nursing Home Residents (Anrys et al.), by ADRD Status, 2018-2020

**Supplemental Table 6.** Duration of Exposure to Potential Drug Interactions Among Nursing Home Residents (Beers et al.), by ADRD Status, 2018-2020

**Supplemental Table 7.** Duration of Exposure to Potential Drug Interactions Among Nursing Home Residents (Capiau et al.), by ADRD Status, 2018-2020

**Supplemental Figure 1:** Flow diagram of individuals included in the analytic sample.

Abbreviations: NH, nursing home; PY, person-years

**Supplemental Table 1.** Sequentially adjusted model results for prevalence ratio of potential DDIs between NH stays for individuals living with ADRD vs no ADRD (N=485,251 eligible NH-stays).

|  | **Model 1 (base-adjusted) PR (95%CI)** | **Model 2**  **PR (95%CI)** | **Model 3**  **PR (95%CI)** | **Model 4 (lowest AIC)**  **PR (95%CI)** |
| --- | --- | --- | --- | --- |
| ADRD (vs without ADRD) | 1.09 (1.08, 1.10) | 1.08 (1.08, 1.09) | 1.09 (1.09, 1.10) | 1.09 (1.08, 1.09) |
| Female sex (vs male) | 1.18 (1.17, 1.18) | 1.18 (1.17, 1.18) | 1.18 (1.17, 1.18) | 1.18 (1.17, 1.18) |
| Race/Ethnicity: Asian/Pacific Islander (vs White) | 0.69 (0.67, 0.71) | 0.69 (0.67, 0.71) | 0.69 (0.68, 0.71) | 0.69 (0.68, 0.71) |
| Race/Ethnicity: Black (vs White) | 0.79 (0.79, 0.80) | 0.80 (0.79, 0.81) | 0.79 (0.79, 0.80) | 0.80 (0.79, 0.81) |
| Race/Ethnicity: Hispanic (vs White) | 0.86 (0.85, 0.87) | 0.86 (0.85, 0.88) | 0.86 (0.85, 0.88) | 0.87 (0.85, 0.88) |
| Race/Ethnicity: Unknown/Other (vs White) | 0.86 (0.84, 0.88) | 0.87 (0.85, 0.89) | 0.86 (0.84, 0.88) | 0.87 (0.85, 0.89) |
| Age at start of NH stay (per 5 years) | 0.97 (0.97, 0.97) | NA | 0.97 (0.97, 0.97) | NA |
| Combined comorbidity score (per 1 unit increase) | 1.00 (1.00, 1.00) | 1.00 (1.00, 1.00) | NA | NA |
| Age at start of NH stay (linear spline term) | NA | 1.00 (0.99, 1.00) | NA | 1.00 (0.99, 1.00) |
| Age at start of NH stay (restricted cubic spline term 2) | NA | 0.99 (0.98, 1.00) | NA | 0.99 (0.98, 1.00) |
| Age at start of NH stay (restricted cubic spline term 3) | NA | 0.81 (0.76, 0.86) | NA | 0.81 (0.76, 0.87) |
| Combined comorbidity score (linear spline term) | NA | NA | 0.97 (0.96, 0.97) | 0.97 (0.96, 0.97) |
| Combined comorbidity score (restricted cubic spline term 2) | NA | NA | 1.21 (1.19, 1.23) | 1.21 (1.19, 1.23) |
| Combined comorbidity score (restricted cubic spline term 3) | NA | NA | 0.62 (0.60, 0.65) | 0.62 (0.60, 0.65) |

*Abbreviations: ADRD, Alzheimer’s disease and related dementias; AIC - Akaike Information Criterion; NH, nursing home; PR, Prevalence Ratio (ADRD vs no ADRD)*

*NA – not estimated in that model.*

**Supplemental Table 2.** Prevalence of Potential Drug Interactions Among Nursing Home Residents (Anrys et al.), by ADRD Status, 2018-2020.

| **Potential DDI** | **ADRD n = 340,024** | | **No ADRD n = 145,227** | | **Difference in prevalence, %** | **Estimated Excess Residents with DDI (ADRD Group)** |
| --- | --- | --- | --- | --- | --- | --- |
|  | Residents with DDI, No. | Residents with DDI,  % (95% CLs) | Residents with DDI, No. | Residents with DDI,  % (95% CLs) |  |  |
| Concomitant use of ≥3 CNS-active drugs* | 100767 | 29.64 (29.48, 29.79) | 30813 | 21.22 (21.01, 21.43) | 8.42 | 28624 |
| SSRI + another serotonergic drug (including tramadol) | 56392 | 16.58 (16.46, 16.71) | 16265 | 11.20 (11.04, 11.36) | 5.39 | 18310 |
| SSRI + loop or thiazide diuretic | 51268 | 15.08 (14.96, 15.20) | 20774 | 14.30 (14.12, 14.49) | 0.77 | 2629 |
| Concomitant prescription of ≥2 anticholinergic drugs | 39368 | 11.58 (11.47, 11.69) | 14004 | 9.64 (9.49, 9.80) | 1.94 | 6580 |
| ACE inhibitor or ARB or a potassium-sparing diuretic + a potassium supplement | 35100 | 10.32 (10.22, 10.43) | 17596 | 12.12 (11.95, 12.29) | -1.79 | -6098 |
| Concomitant prescription of ≥2 drugs that reduce potassium (e.g., beta-2 agonists, thiazides, loop diuretics, corticosteroids) | 30087 | 8.85 (8.75, 8.94) | 20138 | 13.87 (13.69, 14.05) | -5.02 | -17063 |
| Concomitant use of ≥2 potassium-sparing drugs (i.e., amiloride, triamterene, eplerenone, spironolactone, ACE inhibitors, ARBs, NSAIDs, trimethoprim/ sulfamethoxazole) | 29542 | 8.69 (8.59, 8.78) | 16004 | 11.02 (10.86, 11.18) | -2.33 | -7929 |
| Acetylcholinesterase inhibitor + a drug that reduces heart rate (antiarrhythmic drugs, beta-blockers, verapamil, or diltiazem) | 36799 | 10.82 (10.72, 10.93) | 1637 | 1.13 (1.07, 1.18) | 9.70 | 32966 |
| Oral NSAID + SSRI or SNRIs | 14444 | 4.25 (4.18, 4.32) | 5773 | 3.98 (3.88, 4.08) | 0.27 | 928 |
| Diuretic + oral NSAID | 11493 | 3.38 (3.32, 3.44) | 7113 | 4.90 (4.79, 5.01) | -1.52 | -5161 |
| ACE inhibitor or ARB + an oral NSAID | 11576 | 3.40 (3.34, 3.47) | 6508 | 4.48 (4.38, 4.59) | -1.08 | -3661 |
| Apixaban + a P-gp inhibitor or a CYP3A4-inhibitor | 11056 | 3.25 (3.19, 3.31) | 6128 | 4.22 (4.12, 4.32) | -0.97 | -3292 |
| Beta-blocker + verapamil or diltiazem | 10322 | 3.04 (2.98, 3.09) | 5581 | 3.84 (3.74, 3.94) | -0.81 | -2745 |
| Atorvastatin or simvastatin or lovastatin + verapamil or diltiazem | 8266 | 2.43 (2.38, 2.48) | 4350 | 3.00 (2.91, 3.08) | -0.56 | -1919 |
| Calcium channel blocker + a CYP3A4 inhibitor | 8376 | 2.46 (2.41, 2.52) | 3938 | 2.71 (2.63, 2.80) | -0.25 | -844 |
| Digoxin + thiazide or loop diuretic | 6828 | 2.01 (1.96, 2.06) | 3579 | 2.46 (2.39, 2.55) | -0.46 | -1552 |
| Simvastatin + amlodipine | 6708 | 1.97 (1.93, 2.02) | 2996 | 2.06 (1.99, 2.14) | -0.09 | -307 |
| Atorvastatin or simvastatin or lovastatin + amiodarone | 5644 | 1.66 (1.62, 1.70) | 3349 | 2.31 (2.23, 2.38) | -0.65 | -2197 |
| Oral anticoagulant + an antiplatelet drug (including aspirin) | 4624 | 1.36 (1.32, 1.40) | 2669 | 1.84 (1.77, 1.91) | -0.48 | -1625 |
| Oral anticoagulant (i.e., vitamin k antagonist or factor Xa inhibitor or direct thrombin inhibitor) + an oral NSAID | 3819 | 1.12 (1.09, 1.16) | 2404 | 1.66 (1.59, 1.72) | -0.53 | -1810 |
| Alprazolam or diazepam or midazolam or triazolam or zolpidem or zopiclone + a CYP3A4 inhibitor | 3776 | 1.11 (1.08, 1.15) | 1976 | 1.36 (1.30, 1.42) | -0.25 | -850 |
| Oral or parenteral corticosteroid + an oral NSAID | 3252 | 0.96 (0.92, 0.99) | 2180 | 1.50 (1.44, 1.57) | -0.54 | -1852 |
| Rivaroxaban + a P-gp inhibitor or a CYP3A4-inhibitor (ketoconazole, itraconazole, voriconazole, posaconazole, fluconazole, diltiazem, verapamil, quinidine, dronedarone, amiodarone, cyclosporine, clarithromycin, erythromycin, or ritonavir) | 3185 | 0.94 (0.90, 0.97) | 1778 | 1.22 (1.17, 1.28) | -0.29 | -978 |
| Vitamin K antagonist + a quinolone | 2851 | 0.84 (0.81, 0.87) | 1789 | 1.23 (1.18, 1.29) | -0.39 | -1338 |
| Antiplatelet drug (including aspirin) + oral NSAID | 2793 | 0.82 (0.79, 0.85) | 1310 | 0.90 (0.85, 0.95) | -0.08 | -274 |
| Digoxin + verapamil or diltiazem | 2339 | 0.69 (0.66, 0.72) | 1208 | 0.83 (0.79, 0.88) | -0.14 | -489 |
| Vitamin K antagonist + amiodarone | 1244 | 0.37 (0.35, 0.39) | 1074 | 0.74 (0.70, 0.78) | -0.37 | -1271 |
| Vitamin K antagonist + trimethoprim/sulfamethoxazole | 1297 | 0.38 (0.36, 0.40) | 914 | 0.63 (0.59, 0.67) | -0.25 | -843 |
| Vitamin K antagonist + a macrolide | 1108 | 0.33 (0.31, 0.35) | 769 | 0.53 (0.49, 0.57) | -0.20 | -692 |
| MAO inhibitor + an antidepressant | 1291 | 0.38 (0.36, 0.40) | 432 | 0.30 (0.27, 0.33) | 0.08 | 280 |
| Digoxin + amiodarone | 644 | 0.19 (0.18, 0.20) | 397 | 0.27 (0.25, 0.30) | -0.08 | -286 |
| Digoxin + some macrolides (i.e., erythromycin, clarithromycin, azithromycin, roxithromycin, or telithromycin) | 697 | 0.21 (0.19, 0.22) | 332 | 0.23 (0.20, 0.25) | -0.02 | -80 |
| Statin + gemfibrozil | 392 | 0.12 (0.10, 0.13) | 243 | 0.17 (0.15, 0.19) | -0.05 | -177 |
| Vitamin K antagonist + a fibrate | 278 | 0.08 (0.07, 0.09) | 228 | 0.16 (0.14, 0.18) | -0.08 | -256 |
| Vitamin K antagonist + metronidazole | 261 | 0.08 (0.07, 0.09) | 172 | 0.12 (0.10, 0.14) | -0.04 | -142 |
| Fluoxetine + tricyclic antidepressant | 249 | 0.07 (0.06, 0.08) | 156 | 0.11 (0.09, 0.13) | -0.03 | -116 |
| Atorvastatin or simvastatin or lovastatin + some macrolides (i.e., erythromycin, clarithromycin, roxithromycin, or telithromycin) | 214 | 0.06 (0.05, 0.07) | 115 | 0.08 (0.07, 0.10) | -0.02 | -55 |
| MAO inhibitor + sympathomimetic drug | 240 | 0.07 (0.06, 0.08) | 87 | 0.06 (0.05, 0.07) | 0.01 | 36 |
| Dabigatran + a P-gp inhibitor (ketoconazole, itraconazole, verapamil, quinidine, amiodarone, dronedarone, cyclosporine, clarithromycin, erythromycin, or ritonavir) | 175 | 0.05 (0.04, 0.06) | 119 | 0.08 (0.07, 0.10) | -0.03 | -104 |
| Lithium + diuretic | 190 | 0.06 (0.05, 0.06) | 62 | 0.04 (0.03, 0.05) | 0.01 | 45 |
| Methotrexate + trimethoprim | 136 | 0.04 (0.03, 0.05) | 80 | 0.06 (0.04, 0.07) | -0.02 | -51 |
| Lithium + ACE inhibitor or an ARB | 150 | 0.04 (0.04, 0.05) | 48 | 0.03 (0.02, 0.04) | 0.01 | 38 |
| Theophylline + a quinolone | 81 | 0.02 (0.02, 0.03) | 82 | 0.06 (0.04, 0.07) | -0.03 | -111 |
| Carbamazepine + verapamil or diltiazem | 106 | 0.03 (0.03, 0.04) | 43 | 0.03 (0.02, 0.04) | 0 | 5 |
| Tamoxifen + citalopram or escitalopram | 99 | 0.03 (0.02, 0.04) | 42 | 0.03 (0.02, 0.04) | 0 | 1 |
| Lithium + NSAID | 54 | 0.02 (0.01, 0.02) | 17 | 0.01 (0.01, 0.02) | 0 | 14 |
| MAO-A inhibitor or nonselective MAO inhibitor + levodopa | 50 | 0.01 (0.01, 0.02) | 14 | 0.01 (0.01, 0.02) | 0.01 | 17 |
| Digoxin + propafenone | 38 | 0.01 (0.01, 0.02) | 20 | 0.01 (0.01, 0.02) | 0 | -9 |
| Tamoxifen + vitamin K antagonist | 24 | 0.01 (0, 0.01) | 25 | 0.02 (0.01, 0.03) | -0.01 | -35 |
| Digoxin + quinidine | ** | ** | ** | ** | ** | ** |
| Tamoxifen + paroxetine or fluoxetine or bupropion | 23 | 0.01 (0, 0.01) | 16 | 0.01 (0.01, 0.02) | 0 | -14 |
| Furosemide + ethacrynic acid | 15 | 0 (0, 0.01) | 11 | 0.01 (0, 0.01) | 0 | -11 |
| Vitamin K antagonist + cimetidine | ** | ** | ** | ** | ** | ** |
| Thiopurines + allopurinol | ** | ** | ** | ** | ** | ** |
| Phosphodiesterase type 5-inhibitor + nitrate | ** | ** | ** | ** | ** | ** |
| Carbamazepine + some macrolides (i.e., erythromycin, clarithromycin, roxithromycin, or telithromycin) | ** | ** | ** | ** | ** | ** |
| Edoxaban + a P-gp inhibitor (ketoconazole, itraconazole, verapamil, quinidine, amiodarone, dronedarone, cyclosporine, clarithromycin, erythromycin, or ritonavir) | ** | ** | ** | ** | ** | ** |
| Theophylline + some macrolides (i.e., erythromycin, clarithromycin, roxithromycin, or telithromycin) | ** | ** | ** | ** | ** | ** |
| Ergot alkaloid + some macrolides (i.e., erythromycin, clarithromycin, roxithromycin, or telithromycin) | ** | ** | ** | ** | ** | ** |
| Disopyramide + some macrolides (i.e., erythromycin, clarithromycin, or telithromycin) | 0 | 0 (0, 0) | 0 | 0 (0, 0) | 0 | 0 |
| Procainamide + amiodarone | 0 | 0 (0, 0) | 0 | 0 (0, 0) | 0 | 0 |
| Procainamide + trimethoprim | 0 | 0 (0, 0) | 0 | 0 (0, 0) | 0 | 0 |
| MAO inhibitor + some opioids (i.e., meperidine or fentanyl) | 0 | 0 (0, 0) | 0 | 0 (0, 0) | 0 | 0 |
| Theophylline + cimetidine | 0 | 0 (0, 0) | 0 | 0 (0, 0) | 0 | 0 |
| Theophylline + fluvoxamine | 0 | 0 (0, 0) | 0 | 0 (0, 0) | 0 | 0 |
| Cyclosporine + rifampin | 0 | 0 (0, 0) | 0 | 0 (0, 0) | 0 | 0 |
| *Note: Residents contributed 272,780 person-years of follow-up time. Abbreviations: ACE, angiotensin-converting enzyme; ARB, angiotensin receptor blocker; CLs, confidence limits; CYP, cytochrome P450; DDI, drug interaction; MAO, monoamine oxidase; NSAID, nonsteroidal anti-inflammatory drug; P-gp, P-glycoprotein; Q1, first quartile; Q3, third quartile; SNRI, serotonin and norepinephrine reuptake inhibitor; SSRI, selective serotonin reuptake inhibitor.*  *** Suppressed due to small cell size.* | | | | | | |

**Supplemental Table 3.** Prevalence of Potential Drug Interactions Among Nursing Home Residents (2023 Beers Criteria), by ADRD Status, 2018-2020.

| **Potential DDI** | **ADRD n = 340,024** | | **No ADRD n = 145,227** | | **Difference in prevalence, %** | **Estimated Excess Residents with DDI (ADRD Group)** |
| --- | --- | --- | --- | --- | --- | --- |
|  | Residents with DDI, No. | Residents with DDI,  % (95% CLs) | Residents with DDI, No. | Residents with DDI,  % (95% CLs) |  |  |
| Any combination of ≥3 CNS-active drugs | 71671 | 21.08 (20.94, 21.22) | 24565 | 16.91 (16.72, 17.11) | 4.16 | 14156 |
| Concomitant use of ≥2 medications with anticholinergic properties | 39368 | 11.58 (11.47, 11.69) | 14004 | 9.64 (9.49, 9.80) | 1.94 | 6580 |
| Opioids + benzodiazepines | 34487 | 10.14 (10.04, 10.24) | 14595 | 10.05 (9.90, 10.21) | 0.09 | 315 |
| Opioids + gabapentin or pregabalin | 25584 | 7.52 (7.44, 7.61) | 17337 | 11.94 (11.77, 12.11) | -4.41 | -15008 |
| Warfarin + amiodarone or ciprofloxacin or macrolides (excluding azithromycin) or trimethoprim-sulfamethoxazole or SSRIs | 8833 | 2.60 (2.54, 2.65) | 5194 | 3.58 (3.48, 3.67) | -0.98 | -3328 |
| Peripheral alpha-1 blockers + loop diuretics* | 2976 | 1.29 (1.25, 1.34) | 1588 | 1.66 (1.58, 1.74) | -0.37 | -1256 |
| Concomitant use of ≥2 RAS inhibitors or potassium-sparing diuretics | 2790 | 0.82 (0.79, 0.85) | 1761 | 1.21 (1.16, 1.27) | -0.39 | -1333 |
| Phenytoin + trimethoprim-sulfamethoxazole | 218 | 0.06 (0.06, 0.07) | 74 | 0.05 (0.04, 0.06) | 0.01 | 45 |
| Lithium + ACEIs or ARBs or ARNIs | 150 | 0.04 (0.04, 0.05) | 48 | 0.03 (0.02, 0.04) | 0.01 | 38 |
| Lithium + loop diuretics | 138 | 0.04 (0.03, 0.05) | 49 | 0.03 (0.03, 0.04) | 0.01 | 23 |
| Theophylline + ciprofloxacin | 27 | 0.01 (0.01, 0.01) | 32 | 0.02 (0.02, 0.03) | -0.01 | -48 |
| Theophylline + cimetidine | 0 | 0 | 0 | 0 | 0 | 0 |
| *Note: Residents contributed 272,780 person-years of follow-up time. Abbreviations: ACEIs, angiotensin-converting enzyme inhibitors; ARBs, angiotensin receptor blockers; ARNIs, angiotensin receptor-neprilysin inhibitors; CLs, confidence limits; CNS, central nervous system; DDI, drug interaction; Q1, first quartile; Q3, third quartile; RAS, renin-angiotensin system; SSRIs, selective serotonin reuptake inhibitor. *Calculations were restricted to females (because the 2023 Beers Criteria notes that this potential DDI specifically increases the risk of urinary incontinence in older women) and included both selective and non-selective peripheral alpha-1 blockers (Overall, n = 325,845; with ADRD, n = 230,276; no ADRD, n = 95,569).*  *** Suppressed due to small cell size.* | | | | | | |

**Supplemental Table 4.** Prevalence of Potential Drug Interactions Among Nursing Home Residents (Capiau et al.), by ADRD Status, 2018-2020.

| **Potential DDI** | **ADRD n = 340,024** | | **No ADRD n = 145,227** | | **Difference in prevalence, %** | **Estimated Excess Residents with DDI (ADRD Group)** |
| --- | --- | --- | --- | --- | --- | --- |
|  | Residents with DDI, No. | Residents with DDI, % (95% CLs) | Residents with DDI, No. | Residents with DDI, % (95% CLs) |  |  |
| SSRIs or SNRIs + direct oral anticoagulants (apixaban, dabigatran, edoxaban, or rivaroxaban) | 25739 | 7.57 (7.48, 7.66) | 10388 | 7.15 (7.02, 7.29) | 0.42 | 1417 |
| Antiplatelet drugs + direct oral anticoagulants (apixaban, dabigatran, edoxaban, or rivaroxaban) | 3744 | 1.10 (1.07, 1.14) | 2055 | 1.42 (1.35, 1.48) | -0.31 | -1067 |
| NSAIDs + direct oral anticoagulants (apixaban, dabigatran, edoxaban, or rivaroxaban) | 3131 | 0.92 (0.89, 0.95) | 1876 | 1.29 (1.23, 1.35) | -0.37 | -1261 |
| Anticoagulants + direct oral anticoagulants (apixaban, dabigatran, edoxaban, or rivaroxaban) | 2262 | 0.67 (0.64, 0.69) | 1089 | 0.75 (0.71, 0.80) | -0.08 | -288 |
| Primidone + direct oral anticoagulants (apixaban, dabigatran, edoxaban, or rivaroxaban) | 641 | 0.19 (0.17, 0.20) | 272 | 0.19 (0.17, 0.21) | 0 | 4 |
| Phenytoin + direct oral anticoagulants (apixaban, dabigatran, edoxaban, or rivaroxaban) | 359 | 0.11 (0.09, 0.12) | 148 | 0.10 (0.09, 0.12) | 0 | 12 |
| Carbamazepine + direct oral anticoagulants (apixaban, dabigatran, edoxaban, or rivaroxaban) | 299 | 0.09 (0.08, 0.10) | 102 | 0.07 (0.06, 0.09) | 0.02 | 60 |
| Phenobarbital + direct oral anticoagulants (apixaban, dabigatran, edoxaban, or rivaroxaban) | 70 | 0.02 (0.02, 0.03) | 37 | 0.03 (0.02, 0.04) | 0 | -17 |
| Clarithromycin + direct oral anticoagulants (apixaban, dabigatran, edoxaban, or rivaroxaban) | 61 | 0.02 (0.01, 0.02) | 42 | 0.03 (0.02, 0.04) | -0.01 | -37 |
| Rifampin + direct oral anticoagulants (apixaban, dabigatran, edoxaban, or rivaroxaban) | 52 | 0.02 (0.01, 0.02) | 54 | 0.04 (0.03, 0.05) | -0.02 | -74 |
| Erythromycin + direct oral anticoagulants (apixaban, dabigatran, edoxaban, or rivaroxaban) | 24 | 0.01 (0, 0.01) | 19 | 0.01 (0.01, 0.02) | -0.01 | -20 |
| Verapamil + dabigatran | 16 | 0 (0, 0.01) | 12 | 0.01 (0, 0.01) | 0 | -12 |
| Voriconazole + apixaban or rivaroxaban | 12 | 0 (0, 0.01) | 19 | 0.01 (0.01, 0.02) | -0.01 | -32 |
| Protease inhibitors + direct oral anticoagulants (apixaban, dabigatran, edoxaban, or rivaroxaban) | ** | ** | ** | ** | ** | ** |
| Ketoconazole + direct oral anticoagulants (apixaban, dabigatran, edoxaban, or rivaroxaban) | ** | ** | ** | ** | ** | ** |
| Itraconazole + direct oral anticoagulants (apixaban, dabigatran, edoxaban, or rivaroxaban) | ** | ** | ** | ** | ** | ** |
| Posaconazole + apixaban or rivaroxaban | ** | ** | ** | ** | ** | ** |
| Cyclosporine + dabigatran or edoxaban | 0 | 0 (0, 0) | 0 | 0 (0, 0) | 0 | 0 |
| St. John's wort + direct oral anticoagulants (apixaban, dabigatran, edoxaban, or rivaroxaban) | 0 | 0 (0, 0) | 0 | 0 (0, 0) | 0 | 0 |
| Ginkgo biloba + direct oral anticoagulants (apixaban, dabigatran, edoxaban, or rivaroxaban) | 0 | 0 (0, 0) | 0 | 0 (0, 0) | 0 | 0 |
| *Note: Residents contributed 272,780 person-years of follow-up time. Abbreviations: CLs, confidence limits; DDI, drug interaction; NSAID, nonsteroidal anti-inflammatory drug; Q1, first quartile; Q3, third quartile; SNRI, serotonin and norepinephrine reuptake inhibitor; SSRI, selective serotonin reuptake inhibitor. ** Suppressed due to small cell size.* | | | | | | |

**Supplemental Table 5.** Duration of Exposure to Potential Drug Interactions Among Nursing Home Residents (Anrys et al.), by ADRD Status, 2018-2020.

| **Potential DDI** | **ADRD n = 340,024** | | **No ADRD n = 145,227** | | **Difference in median days** |
| --- | --- | --- | --- | --- | --- |
|  | Residents with DDI, No. | Median Days of DDI Exposure (Q1, Q3) | Residents with DDI, No. | Median Days of DDI Exposure (Q1, Q3) |  |
| Concomitant use of ≥3 CNS-active drugs | 100767 | 41 (12, 148) | 30813 | 28 (9, 112) | 13 |
| SSRI + another serotonergic drug (including tramadol) | 56392 | 49 (15, 157) | 16265 | 34 (13, 120) | 15 |
| SSRI + loop or thiazide diuretic | 51268 | 68 (20, 204) | 20774 | 54 (16, 174) | 14 |
| Concomitant prescription of ≥2 anticholinergic drugs | 39368 | 24 (8, 79) | 14004 | 17 (7, 57) | 7 |
| ACE inhibitor or ARB or a potassium-sparing diuretic + a potassium supplement | 35100 | 52 (14, 175) | 17596 | 41 (13, 145) | 11 |
| Concomitant prescription of ≥2 drugs that reduce potassium | 30087 | 15 (6, 56) | 20138 | 17 (6, 58) | -2 |
| Concomitant use of ≥2 potassium-sparing drugs | 29542 | 17 (7, 65) | 16004 | 18 (7, 61) | -1 |
| Acetylcholinesterase inhibitor + a drug that reduces heart rate (antiarrhythmic drugs, beta-blockers, verapamil, or diltiazem) | 36799 | 81 (24, 236) | 1637 | 83 (28, 230) | -2 |
| Oral NSAID + SSRI or SNRIs | 14444 | 30 (10, 115) | 5773 | 29 (10, 90) | 1 |
| Diuretic + oral NSAID | 11493 | 27 (9, 94) | 7113 | 24 (9, 76) | 3 |
| ACE inhibitor or ARB + an oral NSAID | 11576 | 30 (10, 104) | 6508 | 25 (10, 78) | 5 |
| Apixaban + a P-gp inhibitor or a CYP3A4-inhibitor | 11056 | 40 (11, 146) | 6128 | 33 (10, 125) | 7 |
| Beta-blocker + verapamil or diltiazem | 10322 | 52 (15, 172) | 5581 | 38 (14, 145) | 14 |
| Atorvastatin or simvastatin or lovastatin + verapamil or diltiazem | 8266 | 53 (16, 170) | 4350 | 40.50 (14, 142) | 12.50 |
| Calcium channel blocker + a CYP3A4 inhibitor | 8376 | 13 (4, 59) | 3938 | 12 (4, 50) | 1 |
| Digoxin + thiazide or loop diuretic | 6828 | 51 (15, 161) | 3579 | 40 (14, 146) | 11 |
| Simvastatin + amlodipine | 6708 | 53 (17, 175) | 2996 | 35 (14, 132) | 18 |
| Atorvastatin or simvastatin or lovastatin + amiodarone | 5644 | 48 (15, 149) | 3349 | 37 (13, 123) | 11 |
| Oral anticoagulant + an antiplatelet drug (including aspirin) | 4624 | 35 (12, 113) | 2669 | 29 (10, 93) | 6 |
| Oral anticoagulant (i.e., vitamin k antagonist or factor Xa inhibitor or direct thrombin inhibitor) + an oral NSAID | 3819 | 19 (7, 65) | 2404 | 18 (7, 54) | 1 |
| Alprazolam or diazepam or midazolam or triazolam or zolpidem or zopiclone + a CYP3A4 inhibitor | 3776 | 16 (6, 58) | 1976 | 17 (6, 57) | -1 |
| Oral or parenteral corticosteroid + an oral NSAID | 3252 | 7 (5, 22) | 2180 | 9 (5, 22) | -2 |
| Rivaroxaban + a P-gp inhibitor or a CYP3A4-inhibitor (ketoconazole, itraconazole, voriconazole, posaconazole, fluconazole, diltiazem, verapamil, quinidine, dronedarone, amiodarone, cyclosporine, clarithromycin, erythromycin, or ritonavir) | 3185 | 37 (10, 140) | 1778 | 27 (9, 111) | 10 |
| Vitamin K antagonist + a quinolone | 2851 | 7 (4, 10) | 1789 | 7 (5, 10) | 0 |
| Antiplatelet drug (including aspirin) + oral NSAID | 2793 | 24 (8, 85) | 1310 | 25 (10, 74) | -1 |
| Digoxin + verapamil or diltiazem | 2339 | 56 (16, 190) | 1208 | 34 (12, 124) | 22 |
| Vitamin K antagonist + amiodarone | 1244 | 38 (14, 123.50) | 1074 | 29 (12, 87) | 9 |
| Vitamin K antagonist + trimethoprim/sulfamethoxazole | 1297 | 7 (5, 10) | 914 | 7 (5, 11) | 0 |
| Vitamin K antagonist + a macrolide | 1108 | 5 (4, 6) | 769 | 5 (4, 6) | 0 |
| MAO inhibitor + an antidepressant | 1291 | 10 (6, 27) | 432 | 10 (6, 17) | 0 |
| Digoxin + amiodarone | 644 | 30 (11, 93.50) | 397 | 23 (8, 69) | 7 |
| Digoxin + some macrolides (i.e., erythromycin, clarithromycin, azithromycin, roxithromycin, or telithromycin) | 697 | 5 (4, 6) | 332 | 5 (4, 6) | 0 |
| Statin + gemfibrozil | 392 | 37 (14, 120.50) | 243 | 33 (14, 133) | 4 |
| Vitamin K antagonist + a fibrate | 278 | 43.50 (14, 131) | 228 | 35 (13, 92.50) | 8.50 |
| Vitamin K antagonist + metronidazole | 261 | 7 (4, 10) | 172 | 7 (4, 11) | 0 |
| Fluoxetine + tricyclic antidepressant | 249 | 41 (16, 125) | 156 | 50 (20.50, 107.50) | -9 |
| Atorvastatin or simvastatin or lovastatin + some macrolides (i.e., erythromycin, clarithromycin, roxithromycin, or telithromycin) | 214 | 7 (5, 14) | 115 | 10 (7, 14) | -3 |
| MAO inhibitor + sympathomimetic drug | 240 | 15.50 (7, 63.50) | 87 | 11 (5, 41) | 4.50 |
| Dabigatran + a P-gp inhibitor (ketoconazole, itraconazole, verapamil, quinidine, amiodarone, dronedarone, cyclosporine, clarithromycin, erythromycin, or ritonavir) | 175 | 40 (14, 155) | 119 | 29 (12, 107) | 11 |
| Lithium + diuretic | 190 | 41 (13, 135) | 62 | 45 (14, 139) | -4 |
| Methotrexate + trimethoprim | 136 | 7 (5, 11) | 80 | 7.50 (5, 11.50) | -0.50 |
| Lithium + ACE inhibitor or an ARB | 150 | 48.50 (19, 166) | 48 | 24 (5.50, 100) | 24.50 |
| Theophylline + a quinolone | 81 | 7 (5, 9) | 82 | 7 (5, 10) | 0 |
| Carbamazepine + verapamil or diltiazem | 106 | 45 (15, 181) | 43 | 31 (14, 129) | 14 |
| Tamoxifen + citalopram or escitalopram | 99 | 82 (30, 207) | 42 | 57 (21, 211) | 25 |
| Lithium + NSAID | 54 | 45.50 (12, 105) | 17 | 41 (28, 94) | 4.50 |
| MAO-A inhibitor or nonselective MAO inhibitor + levodopa | 50 | 8 (6, 13) | 14 | 11.50 (7, 16) | -3.50 |
| Digoxin + propafenone | 38 | 47 (21, 226) | 20 | 24 (12, 81.50) | 23 |
| Tamoxifen + vitamin K antagonist | 24 | 28 (8.50, 73.50) | 25 | 39 (19, 57) | -11 |
| Digoxin + quinidine | ** | ** | ** | ** | ** |
| Tamoxifen + paroxetine or fluoxetine or bupropion | 23 | 43 (15, 119) | 16 | 25.50 (21.50, 106) | 17.50 |
| Furosemide + ethacrynic acid | 15 | 11 (3, 22) | 11 | 8 (4, 22) | 3 |
| Vitamin K antagonist + cimetidine | ** | ** | ** | ** | ** |
| Thiopurines + allopurinol | ** | ** | ** | ** | ** |
| Phosphodiesterase type 5-inhibitor + nitrate | ** | ** | ** | ** | ** |
| Carbamazepine + some macrolides (i.e., erythromycin, clarithromycin, roxithromycin, or telithromycin) | ** | ** | ** | ** | ** |
| Edoxaban + a P-gp inhibitor (ketoconazole, itraconazole, verapamil, quinidine, amiodarone, dronedarone, cyclosporine, clarithromycin, erythromycin, or ritonavir) | ** | ** | ** | ** | ** |
| Theophylline + some macrolides (i.e., erythromycin, clarithromycin, roxithromycin, or telithromycin) | ** | ** | ** | ** | ** |
| Ergot alkaloid + some macrolides (i.e., erythromycin, clarithromycin, roxithromycin, or telithromycin) | ** | ** | ** | ** | ** |
| Theophylline + cimetidine | 0 | 0 (0, 0) | 0 | 0 (0, 0) | 0 |
| Theophylline + fluvoxamine | 0 | 0 (0, 0) | 0 | 0 (0, 0) | 0 |
| Cyclosporine + rifampin | 0 | 0 (0, 0) | 0 | 0 (0, 0) | 0 |
| Disopyramide + some macrolides (i.e., erythromycin, clarithromycin, or telithromycin) | 0 | 0 (0, 0) | 0 | 0 (0, 0) | 0 |
| Procainamide + amiodarone | 0 | 0 (0, 0) | 0 | 0 (0, 0) | 0 |
| Procainamide + trimethoprim | 0 | 0 (0, 0) | 0 | 0 (0, 0) | 0 |
| MAO inhibitor + some opioids (i.e., meperidine or fentanyl) | 0 | 0 (0, 0) | 0 | 0 (0, 0) | 0 |
| *Note: Residents contributed 272,780 person-years of follow-up time. Abbreviations: ACE, angiotensin-converting enzyme; ARB, angiotensin receptor blocker; CLs, confidence limits; CYP, cytochrome P450; DDI, drug interaction; MAO, monoamine oxidase; NSAID, nonsteroidal anti-inflammatory drug; P-gp, P-glycoprotein; Q1, first quartile; Q3, third quartile; SNRI, serotonin and norepinephrine reuptake inhibitor; SSRI, selective serotonin reuptake inhibitor. * Median days of DDI exposure was calculated among those with at least one day of concurrent use for the potential DDI. Days of DDI exposure was not required to be consecutive. ** Suppressed due to small cell size.* | | | | | |

**Supplemental Table 6.** Duration of Exposure to Potential Drug Interactions Among Nursing Home Residents (Beers et al.), by ADRD Status, 2018-2020

| **Potential DDI** | **ADRD n = 340,024** | | **No ADRD n = 145,227** | | **Difference in median days** |
| --- | --- | --- | --- | --- | --- |
|  | Residents with DDI, No. | Median Days of DDI Exposure (Q1, Q3) | Residents with DDI, No. | Median Days of DDI Exposure (Q1, Q3) |  |
| Any combination of ≥3 CNS-active drugs | 71671 | 30 (9, 111) | 24565 | 24 (7, 91) | 6 |
| Concomitant use of ≥2 medications with anticholinergic properties | 39368 | 24 (8, 79) | 14004 | 17 (7, 57) | 7 |
| Opioids + benzodiazepines | 34487 | 8 (3, 26) | 14595 | 9 (4, 27) | -1 |
| Opioids + gabapentin or pregabalin | 25584 | 24 (8, 80) | 17337 | 23 (8, 75) | 1 |
| Warfarin + amiodarone or ciprofloxacin or macrolides (excluding azithromycin) or trimethoprim-sulfamethoxazole or SSRIs | 8833 | 35 (10, 134) | 5194 | 27 (8, 102) | 8 |
| Peripheral alpha-1 blockers + loop diuretics** | 2976 | 49 (15, 161) | 1588 | 38.50 (13, 139) | 10.50 |
| Concomitant use of ≥2 RAS inhibitors or potassium-sparing diuretics | 2790 | 21 (8, 51) | 1761 | 19 (9, 45) | 2 |
| Phenytoin + trimethoprim-sulfamethoxazole | 218 | 7 (5, 12) | 74 | 7 (5, 10) | 0 |
| Lithium + ACEIs or ARBs or ARNIs | 150 | 48.50 (19, 166) | 48 | 24 (5.50, 100) | 24.50 |
| Lithium + loop diuretics | 138 | 35 (12, 121) | 49 | 35 (12, 94) | 0 |
| Theophylline + ciprofloxacin | 27 | 7 (5, 7) | 32 | 7 (4.50, 10) | 0 |
| Theophylline + cimetidine | 0 | 0 (0, 0) | 0 | 0 (0, 0) | 0 |
| *Note: Residents contributed 272,780 person-years of follow-up time. Abbreviations: ACEIs, angiotensin-converting enzyme inhibitors; ARBs, angiotensin receptor blockers; ARNIs, angiotensin receptor-neprilysin inhibitors; CLs, confidence limits; CNS, central nervous system; DDI, drug interaction; Q1, first quartile; Q3, third quartile; RAS, renin-angiotensin system; SSRIs, selective serotonin reuptake inhibitor. * Median days of DDI exposure was calculated among those with at least one day of concurrent use for the potential DDI. Days of DDI exposure was not required to be consecutive. ** Calculations were restricted to females (because the 2023 Beers Criteria notes that this potential DDI specifically increases the risk of urinary incontinence in older women) and included both selective and non-selective peripheral alpha-1 blockers (Overall, n = 325,845; with ADRD, n = 230,276; no ADRD, n = 95,569).* | | | | | |

**Supplemental Table 7.** Duration of Exposure to Potential Drug Interactions Among Nursing Home Residents (Capiau et al.), by ADRD Status, 2018-2020

| **Potential DDI** | **ADRD n = 340,024** | | **No ADRD n = 145,227** | | **Difference in median days** |
| --- | --- | --- | --- | --- | --- |
|  | Residents with DDI, No. | Median Days of DDI Exposure (Q1, Q3) | Residents with DDI, No. | Median Days of DDI Exposure (Q1, Q3) |  |
| SSRIs or SNRIs + direct oral anticoagulants (apixaban, dabigatran, edoxaban, or rivaroxaban) | 25739 | 69 (21, 195) | 10388 | 58 (18, 181) | 11 |
| Antiplatelet drugs + direct oral anticoagulants (apixaban, dabigatran, edoxaban, or rivaroxaban) | 3744 | 34 (12, 112) | 2055 | 28 (10, 92) | 6 |
| NSAIDs + direct oral anticoagulants (apixaban, dabigatran, edoxaban, or rivaroxaban) | 3131 | 20 (7, 66) | 1876 | 18 (7, 55) | 2 |
| Anticoagulants + direct oral anticoagulants (apixaban, dabigatran, edoxaban, or rivaroxaban) | 2262 | 8 (4, 16) | 1089 | 8 (4, 17) | 0 |
| Primidone + direct oral anticoagulants (apixaban, dabigatran, edoxaban, or rivaroxaban) | 641 | 62 (16, 196) | 272 | 55 (19, 190.50) | 7 |
| Phenytoin + direct oral anticoagulants (apixaban, dabigatran, edoxaban, or rivaroxaban) | 359 | 60 (18, 148) | 148 | 52 (14, 220.50) | 8 |
| Carbamazepine + direct oral anticoagulants (apixaban, dabigatran, edoxaban, or rivaroxaban) | 299 | 60 (16, 153) | 102 | 51 (14, 167) | 9 |
| Phenobarbital + direct oral anticoagulants (apixaban, dabigatran, edoxaban, or rivaroxaban) | 70 | 45.50 (14, 152) | 37 | 116 (42, 216) | -70.50 |
| Rifampin + direct oral anticoagulants (apixaban, dabigatran, edoxaban, or rivaroxaban) | 52 | 12.50 (6.50, 27.50) | 54 | 13 (7, 40) | -0.50 |
| Clarithromycin + direct oral anticoagulants (apixaban, dabigatran, edoxaban, or rivaroxaban) | 61 | 9 (7, 13) | 42 | 10 (5, 12) | -1 |
| Erythromycin + direct oral anticoagulants (apixaban, dabigatran, edoxaban, or rivaroxaban) | 24 | 8 (6, 25.50) | 19 | 6 (2, 13) | 2 |
| Voriconazole + apixaban or rivaroxaban | 12 | 12.50 (5, 15) | 19 | 12 (9, 34) | 0.50 |
| Verapamil + dabigatran | 16 | 24 (7.50, 253) | 12 | 41.50 (13, 90) | -17.50 |
| Protease inhibitors + direct oral anticoagulants (apixaban, dabigatran, edoxaban, or rivaroxaban) | ** | ** | ** | ** | ** |
| Ketoconazole + direct oral anticoagulants (apixaban, dabigatran, edoxaban, or rivaroxaban) | ** | ** | ** | ** | ** |
| Itraconazole + direct oral anticoagulants (apixaban, dabigatran, edoxaban, or rivaroxaban) | ** | ** | ** | ** | ** |
| Posaconazole + apixaban or rivaroxaban | ** | ** | ** | ** | ** |
| Cyclosporine + dabigatran or edoxaban | 0 | 0 (0, 0) | 0 | 0 (0, 0) | 0 |
| St. John's wort + direct oral anticoagulants (apixaban, dabigatran, edoxaban, or rivaroxaban) | 0 | 0 (0, 0) | 0 | 0 (0, 0) | 0 |
| Ginkgo biloba + direct oral anticoagulants (apixaban, dabigatran, edoxaban, or rivaroxaban) | 0 | 0 (0, 0) | 0 | 0 (0, 0) | 0 |
| *Note: Residents contributed 272,780 person-years of follow-up time. Abbreviations: CLs, confidence limits; DDI, drug interaction; NSAID, nonsteroidal anti-inflammatory drug; Q1, first quartile; Q3, third quartile; SNRI, serotonin and norepinephrine reuptake inhibitor; SSRI, selective serotonin reuptake inhibitor.*  ** Median days of DDI exposure was calculated among those with at least one day of concurrent use for the potential DDI. Days of DDI exposure was not required to be consecutive. ** Suppressed due to small cell size.* | | | | | |
